# EEG Signal Processing for Biomedical Applications

**DOI:** 10.3390/s22249754

**Published:** 2022-12-13

**Authors:** Yvonne Tran

**Affiliations:** Department of Linguistics, Macquarie University Hearing, Macquarie University, Sydney, NSW 2109, Australia; yvonne.tran@mq.edu.au

## 1. Introduction

Electroencephalography (EEG) signals are used widely in clinical and research settings. Electrical activity generated from large populations of neurons in the brain is measured using scalp-mounted EEG sensors. As a result, we can obtain information regarding brain activity in various cognitive and emotional states. Due to their ability to provide this type of information, EEG signals are used in applications such as monitoring levels of alertness and mental engagement, investigating chronic conditions, and as signals for biofeedback or assistive devices. Innovations in this field have led to advancements in signal processing methods and the development of novel applications ranging from brain–computer interfaces (BCIs) to neuromarketing. EEG signals can be processed in time, frequency, or spatial domains, providing multi-dimensional means to interpret brain activities. Aside from providing invaluable information, EEG signals also have the advantage of capturing complex neural patterns at a high rate of speed. As a reliable, portable, and non-invasive way to measure the electrical activity in the brain, EEG is a central methodology for affordable and practical research and a promising clinical healthcare tool. This Special Issue focuses on EEG signal processing for biomedical engineering applications with original research, communication, and review papers demonstrating broad methodologies and applications. Fifteen papers address various informative themes. These range from examining physical innovations for the development of EEG sensors to studies in clinical populations such as individuals with epilepsy, spinal cord injury, and Amyotrophic Lateral Sclerosis (ALS). In this Special Issue, many novel EEG signal-processing strategies and analysis techniques are explored.

## 2. Overview of Contribution

Two communication papers are included in the Special Issue, with the first highlighting a new concept for EEG sensor development [1]. As EEG signals are acquired from the scalp, this paper presented an anatomically realistic textile-based head phantom for the assessment of EEG sensors. A gelatin-based head phantom is long-lasting and can accurately mimic body electrode frequencies, allowing for stable and accurate measurements of EEG signals. The outcomes from this paper will add to this field by allowing newly developed EEG electrodes to be validated. The second communication paper [2] presented a novel network analysis approach using a multi-layer model. Traditionally, in graph analysis, models are based on single layers. However, with the brain being a multi-layer network, analysis will be constrained when conveying brain topologies through single-layer models. Multi-layer networks produce more reliable approximations of the topology and dynamics of motor functions from the brain.

Within the theme of graph analysis, papers by Hag et al., Perez-Ortiz et al., and Šverko et al. all examined functional connectivity from EEG signals [3,4,5]. Friston (1994) defines functional connectivity as the temporal coincidence of spatially distant neurophysiological events [6]. It is said to have a measurable statistical relationship that captures two things occurring together which are related to each other [7]. Hag et al. used hybrid multi-domain EEG-based machine learning feature sets to assess mental stress. The functional connectivity network showed a statistically significant decrease during mental stress. Results from the time, frequency, and functional connectivity domains showed that the accuracy in detecting mental stress from EEG signals was highest with functional connectivity. However, combining the features from all three domains improved the overall accuracy, demonstrating greater nuance when using multiple EEG processing methods. Perez-Ortiz et al. examined functional connectivity and frequency power alterations in evoked potentials, specifically P300, in patients with ALS. P300 signals were utilized in a BCI device to control a robotic arm. People with ALS had overactivated beta bands and under-activated alpha bands in connectivity measures compared to the control participants. The results indicated that connectivity in EEG signals may be a valuable tool for monitoring disease progress and measuring cognitive atrophy. In their study, Šverko et al. presented a method for analyzing EEG connectivity. In this paper, they proposed the complex Pearson correlation coefficient (CPCC) as a unique single measure to provide information on phase locking and weighted phase lag. This proposed connectivity measure could accelerate the computation of brain connectivity and enhance our understanding of brain processes. A review paper in this issue also showed the importance of connectivity measures in mental stress assessment. In their review [8], Katmah et al. found that the selection of the most appropriate features is crucial to successful mental stress detection. Features with additional connectivity network measures and deep learning approaches could improve detection accuracy in terms of mental stress.

The examination of EEG signals in clinical populations can contribute to a better understanding of brain processes in people with neurological disorders. Tran et al. explored the effects of virtual reality (VR) intervention on the brain activity of people with neuropathic pain and spinal cord injury [9]. A significant reduction in pain intensity was reported after VR intervention, corresponding to statistically significant changes in EEG signals, specifically in the alpha and low gamma bands. Guo and Wang [10] examined brain activity associated with acupuncture. As the scientific explanation for the effects of acupuncture is still unknown, in this research, they studied the power spectrum changes during acupuncture manipulation. They found acupuncture manipulations were associated with delta and alpha rhythms. The neural responses from this study may have implications for the use of acupuncture as a complementary treatment for improving symptoms in neurological disorders. EEG signals in epilepsy were examined in two other studies [11,12], in which novel analysis techniques were assessed. Sánchez-Hernández et al. evaluated dimensionality reduction for feature selection methods with classification methods for epileptic seizures from EEG signals. They found that reducing selected features increased the classifier’s performance. Obukhov et al. used wavelet ridges as a diagnostic EEG feature for the detection of epileptic seizures. It was shown that the application of this methodology will reduce the total duration and number of fragments needed for analysis. Additionally, Hossain et al. examined wavelet decomposition for the correction of movement artifacts in single-channel EEG with fNIRS signals [13]. This method combined wavelet packet decomposition with canonical correlation analysis. This proposed method outperformed comparative methods in removing motion artifacts from a single EEG channel.

Novel EEG signal-processing methods for various applications were also examined in four additional papers which focused on other topics. Zhang et al., in their review paper, discussed the application of transfer learning for EEG signals and BCIs [14]. In machine learning, transfer learning refers to using a model developed for one task as a starting point for constructing another model. The decoding performance in classification and regression tasks was found to be effective with this method. Kamrud et al. [15] investigated the detection of vigilance decrement in both cross-participant and cross-task modes, that is, robust models which can perform in unseen conditions. The research from this paper demonstrated that models could be built for EEG as a marker of vigilance levels even from unseen tasks. Charuthamrong et al. [16] used both auditory- and visual-based event-related potential to assess speech discrimination. Both the visual and auditory methods achieved reasonable accuracy rates and were shown to be potentially suitable for use in an automatic speech discrimination assessment system. Additionally, Zhou et al. used evoked potentials to investigate repetitive transcranial magnetic stimulation (rTMS) [17]. The goal was to develop rTMS EEG-evoked potentials as biomarkers for cortical excitability from rTMS. The changes found in the evoked potentials may have reflected GABAergic-mediated inhibition in specific brain regions.

## 3. Conclusions

A primary focus of this Special Issue was the demonstration of new methods for the analysis of EEG signals for biomedical engineering applications. The examination of various analysis methods led to the presentation of a diverse range of novel strategies. Through their results, the authors of these papers have provided a better understanding of cognitive states and brain activity based on different EEG signal processing methodologies and machine learning strategies.
